# Green and Efficient PEG-Based Ultrasonic-Assisted Extraction of Polysaccharides from Tree Peony Pods and the Evaluation of Their Antioxidant Activity In Vitro

**DOI:** 10.1155/2018/2121385

**Published:** 2018-11-01

**Authors:** Xiaonan Zhang, Qingfeng Ban, Xibo Wang, Zhongjiang Wang

**Affiliations:** Key Laboratory of Soybean Biology in Chinese Ministry of Education, Northeast Agricultural University Harbin 150030, China

## Abstract

We adopted and developed an ultrasonic-assisted extraction method to obtain polysaccharides from tree peony pods using polyethylene glycol (PEG) as the solvent. The technological parameters have been designed as a single factor to enhance the tree peony pod polysaccharide extraction yield. Specific conditions (ultrasound irradiation power, 250 W; ultrasound irradiation time, 30 min; reaction temperature 50°C; liquid-solid ratio, 25 mL/g; and concentration of PEG, 0.2 g/mL) generated an experimental yield of 14.14%  ± 0.44%. Subsequently, the monosaccharide composition of the tree peony pod polysaccharides was determined by HPLC using a 1-phenyl-3-methyl-5-pyrazolone precolumn derivatization method. The results indicated that tree peony pod polysaccharides contained mannoses, rhamnose, glucuronic acid, galacturonic acid, glucose, galactose, arabinose, and fucose with a molar ratio of 1.44 : 2.87 : 0.32 : 18.99 : 3.99 : 10.21 : 0.96 : 1.85 : 0.21. The tree peony pod polysaccharides obtained are mainly galacturonic acid and galactose, which are acidic polysaccharides. Finally, the antioxidant activities (DPPH and FRAP) of the tree peony pod polysaccharides were assessed, and the compounds exhibited moderate antioxidant activities.

## 1. Introduction

Plant polysaccharides are a class of macromolecular compounds that contain more than ten monosaccharides and are polymerized by glycosidic bonds. In recent years, studies have shown the pharmacological effects of polysaccharides, such as antitumor [1, 2], anti-inflammatory [3], antioxidation [4, 5], antiaging [5], and cardiovascular disease inhibition [6]. Because plant polysaccharides are natural compounds and have a wide range of biological activities, research on polysaccharides has attracted more and more attention. In recent years, research on the separation, purification, chemical structure, and pharmacological activities of polysaccharides has gone viral. Tree peony is a ligneous plant that belongs to the family Ranunculaceae [7]. It is an endemic species in China and exists mainly in central and southwestern China. Peony has more than 2,000 years of cultivation history in China, mainly for ornamental and medicinal use [7]. Peony root skin as a traditional Chinese medicine is included in the Pharmacopoeia of the People's Republic of China (TCM) [7]. Peony seed oil was approved by the Chinese government as a new resource food in 2011 [8]. People realize that the demand for peony seed oil is increasing. The large demand for peony seed oil will result in the production of a large number of peony pods. Therefore, research on the components in the pods has gradually gained attention [9]. Peony pods are approximately 60% of the fruit's weight. Pods that are discarded after processing are generally planted or used as low-value fuels. People have been paying more and more attention to the comprehensive use of tree peony resources.

Studies have shown that tree peony pods contain luteolin and apigenin [10], as well as polysaccharides [11]. Tree peony polysaccharides have been reported to have pharmacological effects such as antioxidant [11], hypoglycemic [12], immune regulation [13], and hypolipidemic activities [14]. Choosing the right extraction method depends on the characteristics of the plant material, including the complexity of the material, product cost, environmental effects, and safety. Polysaccharides are natural complex supramolecular compounds that are difficult to extract under mild conditions [15]. At present, the most common method to extract polysaccharides is hot water extraction. However, the disadvantages of hot water extraction are obvious; the compounds are extracted over a long period of time with high extraction temperatures and a low extraction rate, which may lead to polysaccharide degradation during the extraction process [16]. Ultrasound assisted extraction (UAE) is a new technology that has been developed in recent years and has been successfully applied to the extraction of many plant active ingredients [17]. With the advantages of speeding up the extraction efficiency, saving energy, and environmental protection, it is considered a “Green Technology.” UAE has broad application prospects in the extraction of thermally unstable active ingredients and foods that require low temperature processing. With the continuous research and improvements in equipment and technology, UAE will have more extensive application prospects in the fields of food, medicine, and chemical engineering. Ultrasound is a mechanical wave with an effective frequency generally in the range from 20 to 100 kHz. UAE extracts cell contents by the application of the combined effect of cavitations, vibration, pulverization, and agitation generated by ultrasonic waves to destroying cell walls. Thus, choosing the best scheme to improve the extraction efficiency of polysaccharides is critical to reduce the time and energy consumption. PEG solution as a green solvent has the advantages of low price, low flammability, nonvolatile, and low-toxicity [18]. Additionally, PEG is relatively stable at high temperatures and acidity, and a water and organic solvent has better solubility in a redox system [19, 20]. Polyethylene glycol is a polar molecule suitable for ultrasonic energy dissipation [21]. Due to the excellent properties described above, PEG solution is an effective, gentle, and safe extracting solvent compared with traditional and modern solvents. Polyethylene glycol has been used in the extraction of polysaccharides, and its effects are obvious [20, 21].

There is little research focused on the extraction of polysaccharides from tree peony pods; therefore, it is necessary to improve the extraction rate of polysaccharides from peony pod. Additionally, further studies on the bioactivity of polysaccharide extractions with PEG as a solvent are still needed. In this study, PEG solution was used as an extracting solvent to extract tree peony pod polysaccharides under ultrasound irradiation. A single factor combined with response surface methodology was used to optimize the extraction parameters such as the PEG molecular weight, PEG concentration, soaking time, liquid-solid ratio, reaction temperature, ultrasound irradiation power, and ultrasound irradiation time. After deproteinization, decolorization, and desalting of the obtained crude polysaccharides, the glycidic configuration was determined. Finally, the antioxidant activity of the obtained pod polysaccharides was compared in vitro.

## 2. Materials and Methods

### 2.1. Materials and Chemicals

The tree peony fruit was collected in August 2017 from the outskirts of Tongling, Anhui Province, China. After peeling the seeds, they were preserved and dried in a cool and ventilated place. After drying, the preserved pod was crushed with a pulverizer and the 60-80 mesh portion was taken as the experimental material. The moisture content was determined to be 10.2%. The pulverized preserved pod material was stored in a desiccator at a low temperature (4-6°C) and protected from light until it was extracted.

Tripyridyltriazine (TPTZ), 1,1-diphenylpicrylcarbonyl radical (DPPH), 1-phenyl-3-methyl-5-pyrazolone (PMP), and DEAE-cellulose and L-ascorbic acid (Vitamin C, VC) were purchased from Sigma-Aldrich (St. Louis, MO, USA). Polyethylene glycol at molecular weights of 200, 400, 600, 800, and 1000 and papain (E.C. 3.4.22.2; 800 U/mg) was purchased from Shanghai Yuanye Bio-Technology Co Ltd. (Shanghai, China). The monosaccharide D-glucose, D-galactose, D-glucuronic acid, D-galacturonic acid, D-mannose, L-xylose, L-arabinose, L-rhamnose, and L-fucose reference substances were purchased from Shanghai Aladdin Biochemical Technology Co., Ltd. (Shanghai, China). AB-8 macroporous resin (particle diameter 0.3–1.2 mm, surface area 650–700 m2/g, average pore diameter 8.5–9 nm, and cross-linked polystyrene) was purchased from Cangzhou Bon Adsorber Technology Co., Ltd. (Cangzhou, Heibei, China). To eliminate the monomers and porogenic agents from the holes in the macroporous resin during the synthesis procedure, a preliminary adsorbent bead treatment process was performed as follows: the resin was immersed in ethanol solution (95%, v/v) for 24 h at 25°C, washed thoroughly with deionized water until no residue ethanol was present, and finally dried under vacuum prior to use. Ultrapure water used in the solutions and dilutions was obtained from a Milli-Q purification system (Bedford, MA, USA).

### 2.2. Experimental Apparatus

The experimental ultrasonic bath KQ250DB (Kunshan, Jiangsu, China) was used as an extraction device (250 W maximum output power and 40 kHz irradiation frequency). The rectangular bath has a size of 500 × 300 × 150 mm. It can be continuously adjusted through power feedback/control depending on the actual needs for the ultrasound irradiation, ranging from 100 W to 250 W, and maintained through a connection to a constant temperature water bath.

### 2.3. Polysaccharide Extraction Procedure

First, 10.0 g (dry) of accurately preserved fruit was placed it in a 250-mL Erlenmeyer flask. It was extracting in the ultrasonic bath under various conditions to determine the range for the extraction factors and the appropriate values for each factor. Each extraction was filtered, and the filtrate was combined with absolute ethanol to reach an ethanol concentration of 80%. The suspension was stored overnight in a refrigerator at 4°C, after which the floccule precipitates were isolated by centrifugation (8000 × g for 30 min, at 20°C), washed with absolute ethanol three times, and ultimately freeze-dried. The polysaccharide yield was calculated gravimetrically. Each experiment was conducted in triplicate to ensure the accuracy of the experimental results.

### 2.4. Decolorization, Deproteinization, and Desalination

The obtained crude polysaccharides contain many impurities, mainly pigment and protein. Thus, after 2 h of filtering in decoloring solution, we dissolved 5 g of crude polysaccharides in 500 mL ultra-pure water and added AB-8 resin to adjust the solution to 4 pH. We adjusted the pH of the filtrate to 7 using sodium hydroxide solution. The Papain-Sevage method was used to deproteinize the tree peony pod crude polysaccharides based on the procedure by Wang et al. [22] with slight modifications. Briefly, a 10 mg/mL polysaccharide solution was prepared. Then, 50 mL of papain with a polysaccharide mass of 3% was added and the solution was shaken in a water bath at 60°C for 4 h. After enzymolysis, the papain was inactivated at 100°C for 5 min, cooled, and centrifuged at 5000 × g for 15 min and, then, the supernatant was collected. N-butanol and chloroform were mixed in a 1:4 ratio (v/v) to prepare the Sevage reagent, and an appropriate amount was mixed with the supernatant obtained above. After shaking for 20 min, the solution was centrifuged at 3000 × g for 5 min; this was repeated twice. Four volumes of absolute ethanol were added to supernatant, and the mixture was stirred and alcoholized overnight; finally, the polysaccharide was washed with absolute ethanol and acetone and freeze-dried.

Desalination was performed by dialysis. Deionized water was used to prepare a polysaccharide sample solution with a concentration of approximately 10 mg/mL. This sample solution was solubilized by ultrasound and filtered to remove insoluble impurities before the filtrate was loaded into a dialysis bag. Both ends of the dialysis bag were clamped and magnetic dialysis was performed in deionized water for 48 h. The water was changed every 3 hours. After the polysaccharide solution was removed from the dialysis bag, 4 volumes of absolute ethanol were added to it, followed by stirring and drying overnight, filtering, and freeze-drying.

### 2.5. General Analysis of Purified Polysaccharides

The purified polysaccharides were collected for further analysis. The total sugar content was determined by the phenol–sulfuric acid method using glucose as the reference substance [23]. The polysaccharide monosaccharide composition analysis was performed as described by Honda et al. [24]. The monosaccharide composition of the purified polysaccharides was analyzed according to a previously reported method [25].

### 2.6. Determination of DPPH Free Radical Scavenging Capacity

As per a previously described method [26] with slight modifications, 0.1 mL of the 95% ethanol volume fraction from different concentrations of tree peony pod polysaccharides was added to 3.9 mL of 25 mg/L DPPH ethanol solution. After incubation for 30 min in the dark, the absorbance was measured at 517 nm and counted as the Asample. The absorbance determined by replacing the sample with the same volume of the 95% ethanol volume fraction is the Acontrol. The DPPH free radical scavenging ability was expressed as SC% using the following equation: SC% = (1 - Asample/Acontrol) × 100%, where the Acontrol does not contain sample. The absorbance value of the solution at t = 0 and all the other absorbance values were measured 3 times. The results were averaged, and a nonlinear regression curve was fit to obtain the IC50 value.

### 2.7. Determination of Ferric-Reducing Antioxidant Power (FRAP)

The FRAP was determined according to a previously described method [27] with some modifications. First, 25 mL of 300 mM acetate buffer (pH 3.6) was mixed with 2.5 mL of 10 mM TPTZ solutions and 2.5 mL of 20 mM ferric chloride solution. The mixture was maintained at 37°C for 30 min and used as the FRAP solution, Next, 150 *μ*L of tree peony pod polysaccharide sample was added to 2850 *μ*L FRAP solution, which was then incubated in the dark for 30 min. The absorbance was measured at 593 nm, and the result was expressed as the *μ*mol per gram of Trolox (TE)/g.

### 2.8. Statistical Analysis

All experiments were replicated three times. Each replicate is expressed as mean ± SD, and an ANOVA test (using SPSS 17.0) was used to compare the mean values of each treatment. Significant differences between the means of parameters were determined using Duncan's test (p<0.05).

## 3. Results and Discussion

### 3.1. Optimization the Extraction Conditions of Polysaccharides

#### 3.1.1. The Influence of PEG Molecular Weight and Concentration on Peony Pod Polysaccharide Yield

**Figure 1:**
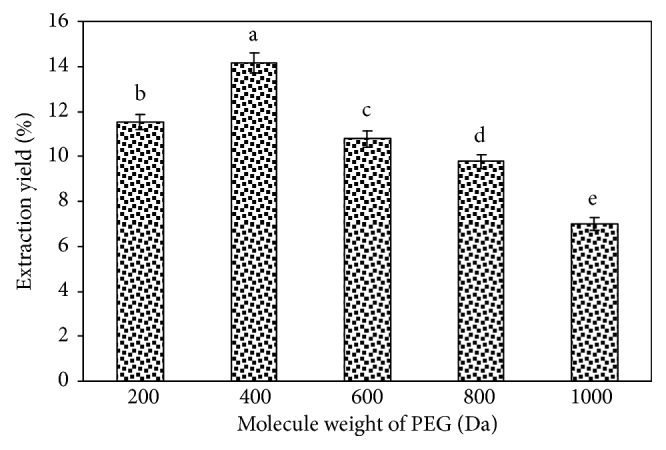
Effects of molecule weight of PEG. Values are mean ± SD (n = 3 replicates). Means that have different letters at the top of each bar are significantly different (P < 0.05).

**Figure 2:**
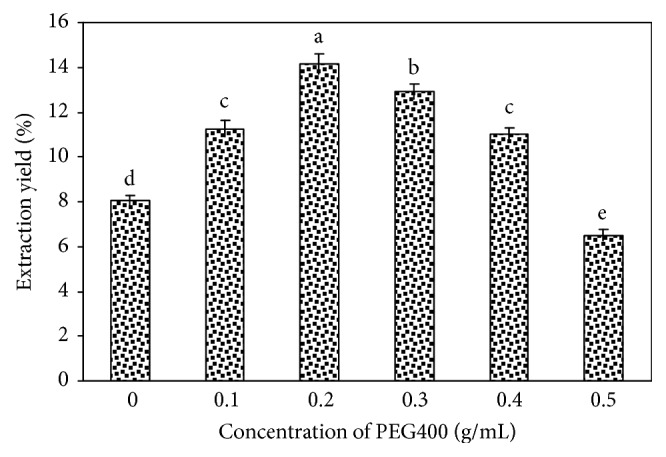
Effects of concentration of PEG400 solution. Values are mean ± SD (n = 3 replicates). Means that have different letters at the top of each bar are significantly different (P < 0.05).

The polarity of water-soluble polyethylene glycol solution depends on the weight of the polyethylene glycol molecule during the extraction process. We examined the molecular weight of polyethylene glycol, and the experimental results are shown in Figure 1. Considering the extraction rate, PEG400 had advantages over the other PEG molecular weights. This illustrates that PEG400 is similar to polar solutions in the literature [20, 28]. Therefore, PEG400 was suitable as the extraction solvent. The polyethylene glycol concentration significantly influences the polarity and viscosity of polyethylene glycol aqueous solutions. From Figure 2, the extraction rate increased when the PEG concentration was 0-0.20 g/mL and decreased when the PEG concentration was 0.20-0.50 g/mL, which is similar to the extraction of polysaccharides from* Nelumbo nucifera* [28]. Thus, 0.20 g/mL PEG400 was adopted for use in this study.

#### 3.1.2. Effect of the Liquid-Solid Ratio on the Tree Peony Pod Polysaccharide Yield

**Figure 3:**
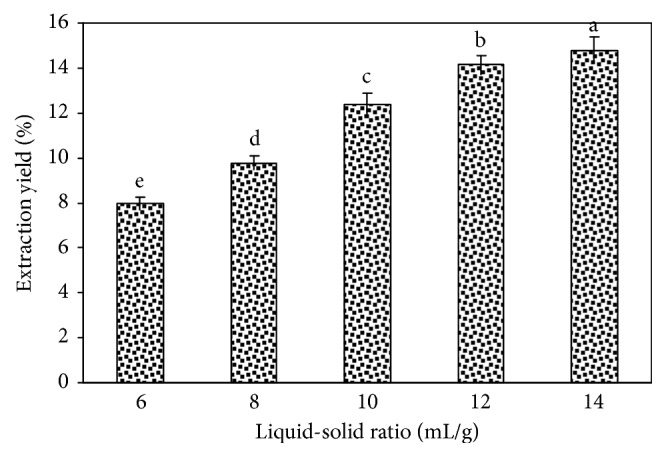
Effect of liquid-solid ratio on the extraction yield of tree peony pod polysaccharides. Values are mean ± SD (n = 3 replicates). Means that have different letters at the top of each bar are significantly different (P < 0.05).

The liquid-solid ratio is another important factor that may affect the yield coefficient of the target product. The effect of this parameter on the tree peony pod polysaccharide yield is shown in Figure 3. We can see that increasing the liquid-solid ratio significantly increased the yield of tree peony pod polysaccharides. Increasing the liquid-solid ratio to 12 mL/g had few effects on the tree peony pod polysaccharide yield coefficient. At this point, the tree peony pod polysaccharide yield stabilized. Thus, a liquid-solid ratio of 12 mL/g was selected as the extraction condition for the tree peony pod polysaccharides.

#### 3.1.3. Effect of Reaction Temperature on the Tree Peony Pod Polysaccharide Yield

**Figure 4:**
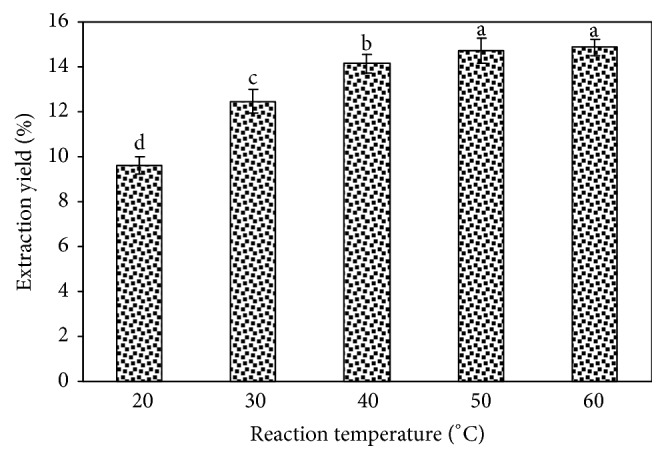
Effect of reaction temperature on the extraction yield of tree peony pod polysaccharides. Values are mean ± SD (n = 3 replicates). Means that have different letters at the top of each bar are significantly different (P < 0.05).

Ultrasound temperature is also an important factor affecting the yield of target plant analytes. As shown in Figure 4, when the reaction temperature is lower than 20°C, the tree peony pod polysaccharide yield is less than 9.61%  ± 0.39%. The tree peony pod polysaccharide yield is positively correlated with the increase in temperature. For example, as the temperature reached 50°C, the tree peony pod polysaccharide yield reached 14.14%  ± 0.44%. Continuously increasing the reaction temperature led to a slow increase in the yield of the tree peony pod polysaccharide. Because the higher temperature is not conducive to the stability of the tree peony pod polysaccharides results, 50°C was selected as the best extraction temperature for the ultrasonic extraction of polysaccharides.

#### 3.1.4. Effect of Ultrasound Irradiation Power on the Tree Peony Pod Polysaccharide Yield

**Figure 5:**
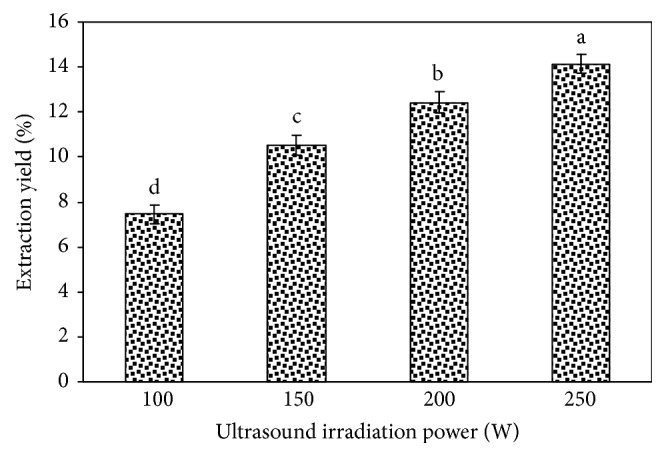
Effect of ultrasound irradiation power on the extraction yield of tree peony pod polysaccharides. Values are mean ± SD (n = 3 replicates). Means that have different letters at the top of each bar are significantly different (P < 0.05).

The effect of ultrasound irradiation power on the tree peony pod polysaccharide yield is shown in Figure 5. We can see from the figure that when other extraction factors are fixed, the polysaccharide yield increases with increasing ultrasound irradiation power. The use of ultrasound mechanical cavitation and thermal effects can increase the media molecule movement speed, thereby increasing the penetration into the media and improving the yield of the active ingredient from the raw material. When the ultrasound irradiation power reached 250 W, the polysaccharide yield was the highest (p < 0.05); thus, 250 W was the best irradiation power for the ultrasound-assisted extraction of tree peony pod polysaccharides.

#### 3.1.5. Effect of Ultrasound Irradiation Time on the Tree Peony Pod Polysaccharide Yield

**Figure 6:**
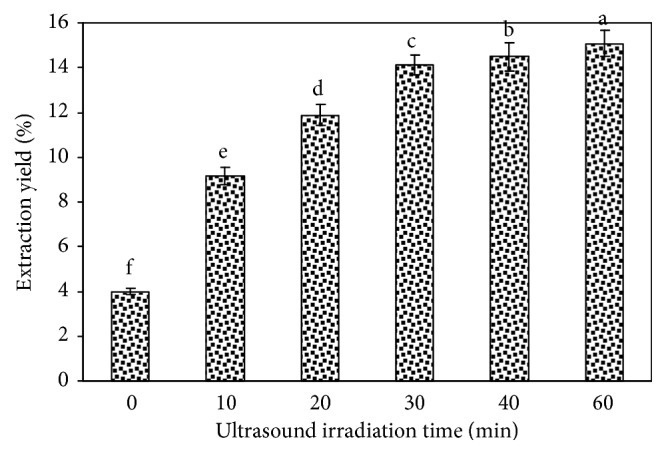
Effect of ultrasound irradiation time on the extraction yield of tree peony pod polysaccharides. Values are mean ± SD (n = 3 replicates). Means that have different letters at the top of each bar are significantly different (P < 0.05).

The effect of different ultrasound irradiation times on the tree peony pod polysaccharide yield is shown in Figure 6. From the figure, as the ultrasonic irradiation time increases, the tree peony pod polysaccharide yield also increases. When the ultrasound irradiation time is 30 min, the increase due to the time shows limited improvement in the tree peony pod polysaccharide yield, and the tree peony pod polysaccharide yield only slightly increases. Therefore, 30 min is the best ultrasound irradiation time for the ultrasound-assisted extraction.

### 3.2. Analysis of the Monosaccharide Composition from Tree Peony Pod Polysaccharides

Under the selected conditions, PMP ramification monosaccharides are isolated from the tree peony pod polysaccharide sample was separated from the baseline. The chromatography peaks were identified based on the retention times in the on-line UV spectrogram, indicating that tree peony pod polysaccharides are composed of mannoses, rhamnose, glucuronic acid, galacturonic acid, glucose, galactose, arabinose, and fucose with a molar ratio of 1.44  : 2.87  : 0.32  : 18.99  : 3.99  : 10.21  : 0.96  : 1.85  : 0.21. The results show that the obtained tree peony pod polysaccharides are mainly galacturonic acid and galactose, which are acidic polysaccharides.

### 3.3. *In Vitro* Antioxidant Activity

#### 3.3.1. DPPH Free Radical Scavenging Capacity

**Figure 7:**
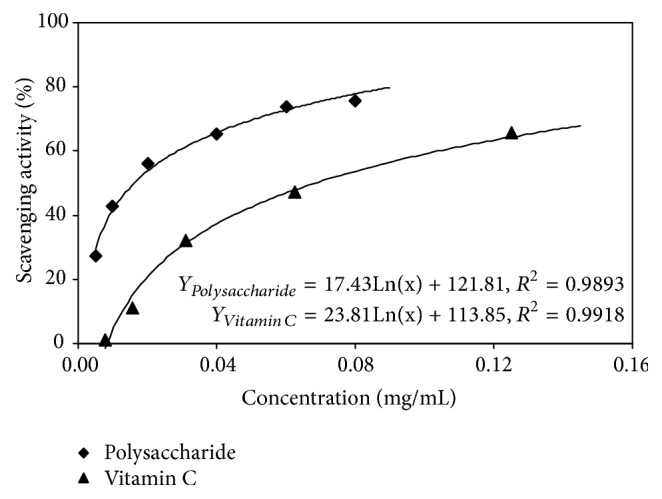
Evaluation of DPPH radical-scavenging activity of tree peony pod polysaccharides.

DPPH reagent was purple-red in methanol and had the strongest absorption at a wavelength of 517 nm. DPPH is structurally stable and can combine with antioxidant hydrogen atoms and electrons and fade. Evaluation of the antioxidant DPPH scavenging capacity was quantitatively tested according to the degree of the color change by the purple-red solution caused by DPPH supplied by antioxidants. The concentration of DPPH is proportional to the absorbance of the solution. The higher the absorbance, the weaker the ability of the antioxidant to scavenge DPPH, and the weaker the antioxidant capacity of the tested substance. The DPPH scavenging capacity of tree peony pod polysaccharides and vitamin C is shown in Figure 7. When the vitamin C concentration is the same as the tree peony pod polysaccharides, the DPPH scavenging ability of the tree peony pod polysaccharides is significantly higher than that of vitamin C. Increasing the tree peony pod polysaccharides concentration increased their scavenging capacity. When the polysaccharide concentration was 0.08 mg/mL, the DPPH clearance rate of the tree peony pod polysaccharides was 75.5%. In contrast, when the vitamin C concentration reached 0.125 mg/mL, its DPPH scavenging capacity was only 65.5%. Therefore, the tree peony pod polysaccharides had a strong scavenging ability for DPPH.

#### 3.3.2. Ferric-Reducing Antioxidant Power (FRAP)

**Figure 8:**
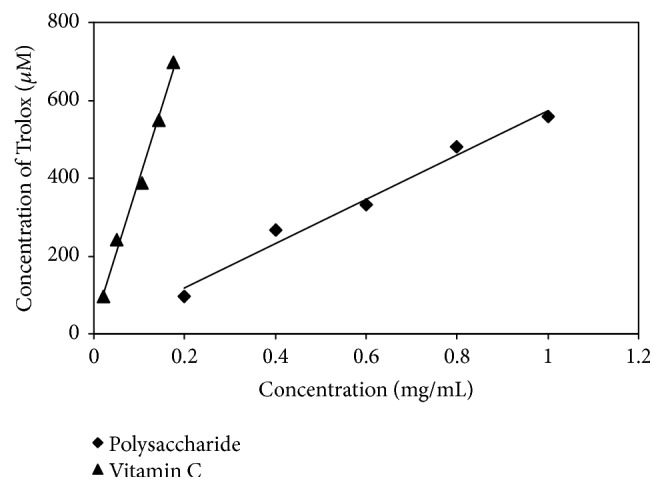
Evaluation of ferric reducing antioxidant power of tree peony pod polysaccharides.

Fe^3+^ in the tripyridyltriazine (TPTZ) reagent can be reduced to Fe^2+^ by antioxidants; the solution is blue, and the absorbance is maximum at a wavelength of 593 nm.  Figure 8 showed that tree peony pod polysaccharide FRAP is weaker than that of vitamin C. Increasing the polysaccharide concentration gradually increased tree peony pod polysaccharide FRAP; thus, the antioxidant activity of the tree peony pod polysaccharides was positively correlated with its concentration.

## 4. Conclusions

In this paper, tree peony pod was used as a raw material with PEG400 solution as an extraction solvent for the ultrasound-assisted extraction of polysaccharides. We optimized the single-factor test extraction conditions for tree peony pod polysaccharides (PEG400 dose of 0.2 g/mL, liquid-solid ratio 25 mL/g, reaction temperature 50°C, ultrasound irradiation power 250 W, and ultrasound irradiation time 30 min). Under these conditions, the polysaccharide yield from tree peony pods was 14.14%  ± 0.44%. The tree peony pod polysaccharides are composed of mannoses, rhamnose, glucuronic acid, galacturonic acid, glucose, galactose, arabinose, and fucose with a molar ratio of 1.44  : 2.87  : 0.32  : 18.99  : 3.99  : 10.21  : 0.96  : 1.85  : 0.21. The tree peony pod polysaccharides are acid polysaccharides that are mainly composed of galacturonic acid and galactose. The results from the analysis and evaluation of their antioxidant activities showed that the DPPH free radical scavenging capacity of tree peony pod polysaccharides was significantly higher than that of vitamin C at the same concentration. The FRAP of tree peony pod polysaccharides was lower than that of vitamin C. However, the FRAP of tree peony pod polysaccharides is positively correlated with its concentration.

## Data Availability

The data used to support the findings of this study are included within the article.
